# Outcome of deliveries among adolescent girls at the Yaoundé central hospital

**DOI:** 10.1186/1471-2393-14-102

**Published:** 2014-03-17

**Authors:** Florent Ymele Fouelifack, Theodore Yangsi Tameh, Eta Ngole Mbong, Philip Njotang Nana, Jeanne Hortence Fouedjio, Jovanny Tsuala Fouogue, Robinson Enow Mbu

**Affiliations:** 1 Obstetrics and Gynecology Unit, Yaoundé Central Hospital, Yaoundé, Cameroon; 2 Research, Education and Health Development Group “GARES - Falaise” Dschang – Cameroon, PO Box: 31186, Yaoundé, Cameroon; 3 Department of Obstetrics and Gynecology, Faculty of Medicine and Biomedical Sciences, University of Yaoundé I, Yaoundé, Cameroon; 4 Ministry of Public Health, Yaoundé, Cameroon; 5 Building Bridges, (BBCAM), Yaoundé, Cameroon

**Keywords:** Adolescent pregnancies, Delivery outcomes, Cameroon

## Abstract

**Background:**

Adolescent pregnancies are a growing public health problem in Cameroon. We sought to study the outcome of such pregnancies, in order to inform public health action.

**Methods:**

A cross-sectional analysis of 5997 deliveries which compared the outcome of deliveries in adolescent (10–19 years old) pregnant women registered at the Yaoundé Central Hospital between 2008 and 2010 to that of their non-adolescent adult (≥ 20 years old) counterparts. Variables used for comparison included socio-demographic and obstetric characteristics of parturients, referral status, and maternal and fetal outcomes. Predictors of maternal and of perinatal mortality were determined through binomial logistic modeling.

**Results:**

Adolescent deliveries represented 9.3% (560) of all pregnancies registered. Adolescent pregnancies had significantly higher rates of both gestational duration extremes: preterm as well as post-term deliveries (29.3% versus 24.5%, p = 0.041 OR 1.28 95% CI 1.01-1.62 and 4.9 versus 2.4%, p = 0.014 OR 2.11 95% CI 1.46-3.87 respectively). Both groups did not differ significantly with respect to mean blood loss, rates of cesarean or instrumental deliveries. Adolescent deliveries however required significantly twice as many episiotomies (OR 2.15 95% CI 1.59-2.90). The likelihood of perineal tears in the adolescent group was significantly higher than that in the adult group on assuming episiotomies done would have been tears if they had not been carried out (OR 1.45 95% CI 1.16-1.82). Adolescent parturients had a higher likelihood of apparent fetal death at birth as well as perinatal fetal death after resuscitation efforts (AOR 1.75 95% CI 1.25-2.47 and AOR 1.69 95% CI 1.17-2.45 respectively).Comparisons of pregnancy outcomes between early (10–14 years), middle (15–17 years) and late adolescence (18–19 years) found no significant differences. Predictors of maternal death included having been referred, having had ≥5 deliveries and preterm deliveries. These were also predictors of perinatal death, as well as being a single mother, primiparous, and multiple gestations.

**Conclusions:**

Adolescent pregnancies in Cameroon compared to those in adults are associated with poorer outcomes. There is need for adolescent-specific services to prevent teenage pregnancies as well as interventions to prevent and manage the above mentioned predictors of in-facility maternal and perinatal mortality.

## Background

Adolescence (10–19 years) is a transitional phase of physical and mental development, involving profound biological, social and psychological changes [1]. Like other sub-Saharan African countries, Cameroon has a high rate of adolescent pregnancies. According to the 2011 Demographic and Health Survey (DHS), 23.3% of Cameroonian women are adolescents, 73.9% of who are single and sexually active but the least likely of all women of reproductive age to use modern contraceptives methods (12.2%) [2]. Four-fifths (81%) of Cameroonian women as revealed by the survey have had sexual intercourse before the age of 20 years with mean age of onset of sexual activity of 17 years. As a result fertility rates are high among women in Cameroon especially adolescents (127 per 1000) [2]. At current fertility rates, a woman will give birth to an average to 5.1 children by the end of her reproductive years [2]. In an earlier study carried out in the North of Cameroon in 2004 by Tebeu et al., 27% of deliveries from 1995 to 2004 were from teenage mothers [3].

Adolescent pregnancies as a result of anatomical and physiological immaturity are prone to more maternal complications. Adolescent pregnancies are therefore a public health problem in Cameroon with an urgent need to focus attention on their reproductive health needs. As a prelude to contributing to the improvement of women’s reproductive health in Cameroon especially adolescents mothers, this study was carried out in the Maternity of Cameroon’s largest hospital (Yaoundé Central Hospital), a tertiary and Teaching hospital located in the cosmopolitan city of Yaoundé with the aim of studying the outcome of pregnancies in adolescents mothers compared to those 20 years and older as well as determine the predictors of in-facility maternal and perinatal mortality.

## Methods

A cross-sectional study carried out at the Obstetrics and Gynecology Unit of Yaoundé Central Hospital which reviewed records of deliveries registered at the center between May 2008 and March 2010. Based on Lorenz’s formula for calculating sample size N = p (1-p) (Z_α_/d)^2^ where N = sample size, and assuming a P (national prevalence of adolescent pregnancy in Cameroon) of 26.54% [2], an α of 0.05 and a Z_α_ of 1.96, a minimum sample size of 300 women was required. In order to increase the power of the study all but 44 deliveries (which did not mention parturient’s age) were included in the study (5997 records in all). The following data were retrieved from the delivery records and noted on a structured pre-tested anonymous data collection sheet: parturient’s age (in years), marital status (single or married), gravidity (number of pregnancies), parity (number of deliveries), mode of delivery (categorized nominally into non-instrumental vaginal, instrumental vaginal and cesarean delivery), the amount of blood lost by the parturient during delivery in cubic centimeters (cc), the state of perineum post-partum (intact, episiotomy or torn) and fetal 1st and 5th minute APGAR scores.

### Statistical analyses

Statistical analyses were done with the aid of SPSS 20.0 for Windows statistical software package. Prior to analyses, all continuous data was tested for normality using histogram plots to justify use of parametric statistical tests. Univariate analyses of continuous variables are presented as frequencies, means and standard deviations. Strength of associations between categorical variables was assessed as odds ratios, chi-squared tests (*X*^2^) used to test for differences between proportions and T-tests for differences between means. Predictors were got using binomial logistic modeling around reference categories. All statistical tests are two-sided and considered statistically significant at p < 0.05.

### Ethical considerations

Ethical clearance for the study was obtained from the management of the study site as well as from the review board of the Yaoundé University Faculty of Medicine and Biomedical Sciences. Data collection and handling were done with strict confidentiality.

## Results

Over five thousand (5997) parturients who delivered at the study site from 2008–2010 were included in the study sample, 560 (9.3%) were adolescents. Singleton pregnancies made up the bulk of the study sample (5365 cases, 94.8%) followed by twin pregnancies (295 cases, 5.0%) and triplet pregnancies 0.2% (16 cases).

### Socio-demographic characteristics and referral status of study participants

The socio-demographic characteristics and referral status of the study population are shown on the Table 1. The youngest parturient was aged 13 years and the oldest 49 years with a mean age (± SD) of 27.34 ± 6.03 years (17.78 ± 1.31 years for the adolescent and 28.32 ± 5.44 years for the non-adolescent parturients). The prevalence rate of adolescent deliveries in our sample was 9.3%. Over three quarters (79.4%) of the adolescent parturients were single compared to 50.1% in the non-adolescent group (p < 0.001, OR 3.85 95% CI 3.10-4.77). The proportion of parturients who were referred was significantly (p = 0.020) higher amongst adolescent parturients compared to non-adolescent parturients (6.4% versus 4.3%, OR 1.53 95% CI 1.07-2.20).

**Table 1:** **Socio**-**demographic characteristics and referral status of study participants**

**Variable**		**Adolescent parturients (10–19 years)**	**Non**-**adolescent parturients (≥20 years)**	**Sig. (p**-**value)**	**OR (95% CI)**	**Entire study sample**
		**n (%)**	**n (%)**			**N (%)**
**Study sample**		560 (9.3)	5437 (90.7)			5997 (100.0)
**Age** (years)	*Mean* ± *SD*	17.78 ± 1.31	28.32 ± 5.44	<0.001		27.34 ± 6.03
	*Range*	13-19	20-49			13-49
**Marital status**	*Single*	432 (79.4)	2654 (50.1)	<0.001	3.85 (3.10-4.77)	2759 (47.2)
	*Married*	112 (20.6)	2647 (49.9)			3086 (52.8)
**Occupation**	*Student*	290 (52.6)	1097 (20.5)	<0.001	4.36 (3.63-5.24)	1387 (23.5)
	*Unemployed*	15 (2.7)	197 (3.7)		1.26 (0.73-2.16)	212 (3.6)
	*Employed*	246 (44.6)	4059 (75.8)		1.00	4305 (72.9)
**Referral status**	*Referred*	36 (6.4)	233 (4.3)	0.020	1.53 (1.07-2.20)	269 (4.5)
	*Non*-*referred*	524 (93.6)	5204 (95.7)			5728 (95.5)

### Parity and gestational age of study participants

A comparison of the 2 groups with respect to parity and gestational age is represented on the Table 2. The number of times participants had delivered varied from 0–12 (1–7 in the adolescent group and 0–12 in the non-adolescent group) with a significant difference between the two groups on comparing mean parities (1.90 ± 0.30 versus 2.08 ± 0.37, p <0.001).

**Table 2:** Parity and pregnancy duration in adolescent parturients compared to their adult counterparts

**Variable**		**Adolescent parturients (10–19 years)**	**Non**-**adolescent parturients (≥20 years)**	**Sig. (p**-**value)**	**OR (95% CI)**	**Entire study sample**
		**n (%)**	**n (%)**			**N (%)**
**Gravida**	*Mean* ± *SD*	1.29 ± 0.63	3.13 ± 2.01	<0.001		27.34 ± 6.03
	*Range*	1-5	1-21			1-21
	*Primigravida*	390 (76.5)	1085 (23.4)	<0.001	7.81 (6.28-9.72)	1475 (28.7)
	*2*-*4*	118 (23.1)	2565 (55.4)		1.00	2683 (52.2)
	≥*5*	2 (0.4)	977 (21.1)		0.04 (0.01-0.18)	979 (19.1)
**Parity**	*Mean* ± *SD*	1.90 ± 0.30	2.08 ± 0.37	<0.001		2.28 ± 1.64
	*Range*	1-7	0-12			0-12
	*0* (*primip*)	50(9.8)	148 (3.2)	<0.001	2.91 (2.09-4.07)	198 (3.9)
	*1*-*4*	459 (90.0)	3959 (85.7)		1.00	4418 (86.1)
	≥ *5*	1 (0.2)	515 (11.1)		0.02 (0.002-0.12)	516 (10.1)
**Pregnancy gestation** (weeks)	*Mean* ± *SD*	38.13 ± 3.19	38.46 ± 2.72	0.050		38.43 ± 2.77
	*Range*	28-44	28-46		1.31 (1.04-1.66)	28-46
	*28*-*36*	110 (29.3)	897 (24.5)	0.007	1.00	1007 (25.0)
	*37*-*41*	252 (67.2)	2694 (73.7)		2.11 (1.15-3.87)	2946 (73.1)
	≥*42*	13 (3.5)	66 (1.8)			79 (2.0)
**Referral status**	*Referred*	36 (6.4)	233 (4.3)	0.020	1.53 (1.07-2.20)	269 (4.5)
	*Non*-*referred*	524 (93.6)	5204 (95.7)			5728 (95.5)
**Preterm delivery**	*Yes*	110 (29.3)	897 (24.5)	0.041	1.28 (1.01-1.62)	1007 (25.0)
	*No*	265 (70.3)	2760 (75.5)			3025 (75.0)
**Post**-**term delivery**	*Yes*	13 (4.9)	66 (2.4)	0.014	2.11 (1.46-3.87)	79 (2.6)
	*No*	252 (95.5)	2694 (97.6)			2946 (97.4)

Pregnancy gestational age ranged from 28 to 46 weeks (Table 2). When assessed as means, pregnancies in the adolescent group lasted shorter than pregnancies in the non-adolescent group, an observation of borderline significance (38.13 ± 3.19 versus 38.46 ± 2.72 weeks respectively, p = 0.050). On categorizing gestational age in weeks, adolescents had significantly higher rates of both post-term (≥42 weeks) as well as preterm deliveries (<37 weeks): 4.9% versus 2.4%, p = 0.014, OR 2.11 95% 1.46-3.87 and 29.3% versus 24.5%, p = 0.041, OR 1.28 95% CI 1.01-1.62 respectively.

### Mode of delivery

Cesarean deliveries accounted for 14.8% of all deliveries and vaginal deliveries for 85.2%. There was no significant difference between the age-group specific rates of cesarean and vaginal deliveries (Table 3). Amongst those who delivered vaginally, 1.1% did so through instrumental deliver, with no significant difference between the rates in the adolescent and non-adolescent group (1.5% and 1.1% respectively, p = 0.411) (Table 3). Forceps deliveries accounted for 97.7% of all instrumental deliveries and vacuum extraction for 5.3% with no significant difference in the rates among adolescent parturients compared to their non-adolescent counterparts.

**Table 3:** Maternal outcomes of study participants

**Variable**		**Adolescent parturients (10–19 years)**	**Non**-**adolescent parturients (≥20 years)**	**Sig. (p-value)**	**OR (95% CI)**	**Entire study sample**
		**n (%)**	**n (%)**			**N (%)**
**Mode of delivery**	*Vaginal*	462 (83.4)	4583 (85.4)	0.207	1.16 (0.92-1.47)	5045 (85.2)
	*Cesarean*	92 (16.6)	784 (14.6)			876 (14.8)
**Instrumental vagina delivery**	*Yes*	7 (1.5)	50 (1.1)	0.411	1.40 (0.63-3.09)	57 (1.1)
	*No*	455 (98.5)	4533 (98.9)			4988 (98.9)
**Instrumental delivery types**	*Forceps*	7 (100.0)	47 (94.0)	1.000	n.a	54 (94.7)
	*Vacuum extractor*	0 (0.0)	3 (6.0)			3 (5.3)
**Perineum after delivery**	*Intact*	280 (69.5)	2865 (76.7)	<0.001	1.00	3145 (76.0)
	*Episiotomy*	60 (14.9)	281 (7.5)		2.19 (1.61-2.96)	341 (8.2)
	*Tear*	63 (15.6)	587 (15.7)		1.10 (0.82-1.46)	650 (15.7)
**Episiotomy done**	*Yes*	60 (14.9)	281 (7.5)	<0.001	2.15 (1.59-2.90)	341 (8.2)
	*No*	343 (85.1)	3452 (92.5)			3795 (91.8)
**Perineal tears**	*Yes*	63 (15.6)	587 (15.7)	0.962	0.99 (0.75-1.32)	650 (15.7)
	*No*	340 (84.4)	3146 (84.3)			3486 (84.3)
**Tear degree**	*1st degree*	59 (93.7)	567 (96.6)	0.278	n.a	626 (96.3)
	*2nd degree*	4 (6.3)	19 (9.2)			23 (3.5)
	*3rd degree*	0 (0.0)	1 (0.2)			1 (0.2)
**Blood loss** (**cc**)	*Mean* ± *SD*	494.29 ± 70.48	499.07 ± 102.06	0.391		498.53 ± 99.34
	≤ *500*	344 (98.6)	3310 (98.0)	0.437	0.70 (0.28-1.74)	3654 (98.0)
	>*500*	5 (1.4)	69 (2.0)			74 (2.0)

**n.a**: not applicable.

### State of perineum after delivery

Episiotomies were required in 8.2% of deliveries (Table 3). Adolescent deliveries required significantly twice more episiotomies than non-adolescent deliveries (14.9% versus 7.5%, p <0.001; OR 2.15 95% CI 1.59-2.90).

Perineal tears were observed in 15.7% of all deliveries with surprisingly no significant difference in the rate in adolescents compared to non-adolescents (15.6% and 15.7% respectively, p = 0.962). However on assuming parturients in both groups who had episiotomies would have had a tear had the procedure not been carried out (that is assuming that episiotomies were tears), the difference in the rate of tears between both groups became significant (30.5 and 23.3% for the adolescent and non-adolescent groups respectively, p = 0.001; OR 1.45 95% CI 1.16-1.82) 1st degree tears represented the bulk of all tears (96.3%) and 3rd degree tears the least (0.2%) with no significant difference in the rate of 1st, 2nd and 3rd degree tears between adolescent and non-adolescent parturients (p = 0.338).

### Maternal blood loss during delivery

Mean intrapartum blood loss was 498.53 ± 99.34 cc with no significant difference in the mean blood loss between the adolescent and non-adolescent groups (Table 3). There was equally no difference in the rate of post-partum bleeding (> 500 cc) between the former and the latter groups (1.4 and 2.0% respectively, p = 0.437).

### Fetal vitality

Irrespective of fetal order, the mean Apgar score at the 1st, as well as at the 5th minutes after resuscitation efforts were significantly higher in non-adolescent mothers compared to their adolescent counterparts (7.69 ± 2.38 versus 7.09 ± 2.77, p < 0.001 at 1st minute and 8.94 ± 2.50 versus 8.45 ± 3.06, p < 0.001 at 5th minutes respectively) (Table 4). When stratified however by birth order, the mean Apgar scores at 1st and 5th minutes were significantly higher in non-adolescent fetuses compared to their adolescent counterparts only for the first fetus (7.70 ± 2.39 versus 7.09 ± 2.81, p < 0.001 and 8.90 ± 2.32 versus 8.44 ± 3.11, p < 0.001) and not for the 2nd and third fetuses.

**Table 4:** Fetal vitality postpartum and maternal mortality

**Variable**		**Adolescent parturients (10**–**19 years)**	**Non**-**adolescent parturients (≥20 years)**	**Sig. (2p-value)**	**OR (95% CI)**	**Entire study sample**
		**n (%)**	**n (%)**			**N (%)**
**Apgar score at 1st minute**	*Mean* ± *SD*	7.09 ± 2.77	7.69 ± 2.38	<0.001	n.a	7.63 ± 2.43
**Apgar score at 5th minute**	*Mean* ± *SD*	8.45 ± 3.06	8.94 ± 2.50	<0.001	n.a	8.89 ± 2.56
**Apparent fetal death**^***a***^	*Yes*	63 (12.4)	352 (7.6)	<0.001	1.75^*^ (1.25-2.47)	415 (8.1)
	*No*	445 (87.6)	4251 (92.4)			4696 (91.9)
**Perinatal fetal death**^***b***^	*Yes*	53 (10.4)	292 (6.3)	0.001	1.69^*^ (1.17-2.45)	345 (6.7)
	*No*	456 (89.6)	4312 (93.7)			4768 (93.3)
**Maternal mortality**	*Deaths*	2	43		0.45 (0.11-1.86)	45
	*Maternal mortality rate*^***c***^	400	800	0.435	n.a	800
	*Maternal mortality ratio*^***d***^	415	940		n.a	890

^***a***^***Apparent death***: *Apgar 0*–*3 1st minute after birth*^***b***^***Perinatal death***: *Apgar 0 after resuscitation efforts of at least 5 minutes.*

^***c***^*per 100*,*000 parturientswho delivered during the study period.*

^***d***^*per 100*,*000 live births registered during the study period.*

*Adjusted for occupation, *referral status*, *mode of delivery*, *mode of vaginal delivery and number of gestas* (*singleton versus multiple pregnancies*).

**n.a**: not applicable.

Irrespective of birth order, the odds of apparent fetal death (Apgar 0–3 at 1st minute) at birth was significantly 1.71 times (OR 1.71 95% CI 1.29-2.27) higher in adolescent pregnancies compared to non-adolescent pregnancies.

After resuscitation, the odds of perinatal death (Apgar 0 at 5th minute) in babies born of adolescent mothers was significantly 1.72 times higher than that of their non-adolescent counterparts (OR 1.72 95% CI 1.26-2.34). On stratifying by birth order the odds of apparent and perinatal death was significantly higher only for first fetuses (OR 1.56 95% 1.13-2.18 and OR 1.77 95% CI 1.31-2.40).

On controlling for mother’s employment status, referral status, mode of delivery, mode of vaginal delivery and if pregnancy was a multiple or singleton, the odds of both apparent and perinatal deaths remained significantly higher in babies born of adolescent mothers compared to those born of non-adolescent mothers (AOR 1.75 95% CI 1.25-2.47 and AOR 1.69 95% CI 1.17-2.45 respectively); Table 4.

### Maternal mortality

Of the 5997 women who delivered during the study period 45 cases of maternal deaths were noted, (death rates of 6.9% versus 0.5% in referred cases compared to non-referred, p < 0.001 and 0.04% versus 0.4%, p = 1.00 respectively amongst non-adolescent and adolescent parturients). The maternal mortality rates and ratios by age group are shown on Table 4. Postpartum hemorrhage was the most common cause of death followed by hypertensive and thromboembolic disorders of pregnancy (46.7%, 24.4% and 8.9% respectively). A similar pattern was observed in both the adult and adolescent group.

### Pregnancy outcomes amongst adolescent parturients

Comparisons of pregnancy outcomes between early (10–14 years), middle (15–17 years) and late adolescence (18–19 years) sub-groups are shown on Table 5.

**Table 5:** Adolescent parturients subgroup characteristics and pregnancy outcomes

**Variable**		**Adolescent sub-group (years)**			***X***^***2***^	**Sig. (p-value)**
		**10-14**	**15-17**	**18-19**		
		**n (%)**	**n (%)**	**n (%)**		
		***14* ( *2.5 *%)**	***177* ( *31.6 *%)**	***369 *** **( *65.9 *)**		
**Referral status** (*referred case*)		0 (0.0)	11 (6.2)	25 (6.8)	1.05	0.887
**Pregnancy gestation** (weeks)	*Mean* ± *SD*	35.88 ± 3.90	38.21 ± 3.29	38.16 ± 2.10	n.a	0.128
	*28*-*36*	5 (62.5)	33 (28.9)	72 (28.5)	4.84	0.353
	*37*-*41*	3 (37.5)	78 (68.4)	171 (67.6)		
	≥*42*	0 (0.0)	3 (2.6)	10 (4.0)		
**Preterm delivery**		5 (62.5)	33 (28.9)	72 (28.5)	4.35	0.135
**Post**-**term delivery**		0 (0.0)	3 (3.7)	10 (5.5)	0.55	0.794
**Mode of delivery**	*Vaginal*	12 (85.7)	144 (81.8)	306 (84.1)	0.49	0.845
	*Cesarean*	2 (14.3)	32 (18.2)	58 (15.9)		
**Instrumental vaginal delivery**		0 (0.0)	0 (0.0)	7 (2.3)	3.62	0.255
**Baby**’**s weight at delivery** (grams)	*Mean* ± *SD*	2680.0 ± 637.95	2937.16 ± 655.66	3019.21 ± 641.61	n.a	0.078
	<*2500*	5 (37.5)	29 (16.4)	61 (16.5)	4.02	0.420
	*2500*-*3999*	9 (64.3)	143 (80.8)	300 (81.3)		
	≥*4000*	0 (0.0)	5 (2.8)	8 (2.2)		
**Perineum after delivery**	*Intact*	6 (60.0)	73 (61.3)	201 (73.4)	6.41	0.110
	*Episiotomy*	2 (20.0)	21 (17.6)	37 (13.5)		
	*Tear*	2 (20.0)	25 (21.0)	36 (13.1)		
**Perineal tear degree**	*1st degree*	2 (100.0)	25 (100.0)	32 (88.9)	3.20	0.244
	*2nd degree or worse*	0 (0.0)	0 (0.0)	4 (11.1)		
**Postpartum blood loss** (**cc**)	*Mean* ± *SD*	457.14 ± 113.39	501.36 ± 52.66	492.06 ± 75.94	n.a	0.194
	>*500*	0 (0.0)	2 (1.8)	4 (1.7)	0.13	1.00
**Apgar score at 1st minute**	*Mean* ± *SD*	7.58 ± 2.39	7.05 ± 2.89	7.10 ± 2.72	n.a	0.811
**Apgar score at 5th minute**	*Mean* ± *SD*	8.66 ± 3.08	8.35 ± 3.31	8.49 ± 2.93	n.a	0.869
**Apparent fetal death**^***a***^		1 (8.3)	22 (13.2)	40 (12.2)	0.29	0.921
**Perinatal fetal death**^***b***^		1 (8.3)	21 (12.6)	31 (9.4)	1.26	0.500
**Maternal mortality**	*Deaths*	0 (0.0)	1 (0.6)	1 (0.3)	0.34	0.566
	*Maternal mortality rate*^***c***^	0.0	0.6	0.3	0.34	0.566
	*Maternal mortality ratio*^***d***^	0.0	667	313	n.a	0.455

^**a**^**Apparent death**: Apgar 0–3 1st minute after birth ^**b**^**Perinatal death**: Apgar 0 after resuscictaion efforts of at least 5 minutes.

^**c**^per 100,000 parturients who delivered during the study period.

^**d**^per 100,000 live births registered during the study period.

**n.a**: not applicable.

### Predictors of maternal and of perinatal mortality

On multivariate analyses, predictors of maternal death included having been referred, having had ≥5 deliveries and preterm deliveries (Table 6). These were also significant predictors of perinatal death, as well as were being a single mother, being primiparous, and having multiple gestations (Table 7).

**Table 6:** Multivariate analyses of predictors of maternal death

**Variables**	**N**	**AOR**	**95% CI**	**Sig. (2 sided)**
**Age (years)**				0.137
*Adolescent*: *10*-*19*	300	0.00	0.00-	0.994
*Adult*: >*19* (*ref*.)	2817	1.00		
**Workstatus**				
*Student*	747	0.23	0.02-2.84	0.251
*Unemployed*	98	0.35	0.07-1.75	0.201
*Employed* (*ref*.)	2272	1.00		
**Matrimonial status**				
*Married* (*ref*.)	1460	1.00		
*Single*	1657	0.83	0.31-2.23	0.707
**Referral status**				
Referred case*	*150*	*4.60*	*1.37*-*15.48*	*0.014*
*Not*-*referred case* (*ref*.)	2967	1.00		
**Gravida**				0.879
*1st pregnancy*	910	0.00	0.00-	0.990
*2*-*4th pregnancy* (*ref*.)	1636	1.00		
≥*5th pregnancy*	571	0.58	0.70-4.70	0.611
**Parity**				0.133
*1st delivery*	118	0.01	0.00-	0.996
*2*-*4th delivery* (*ref*.)	2698	1.00		
≥5th delivery*	*301*	*8.64*	*1.05*-*70.79*	*0.045*
**Gestational age** (weeks)				0.094
<37*	*884*	*2.91*	*1.11*-*7.61*	*0.030*
*37*- < *42* (*ref*.)	2178	1.00		
≥*42*	55	0.00	0.00	0.997
**Delivery mode**				
*Vaginal*	3061	1.00		
*Cesarean*	56	0.00	0.00	0.998
**Number of fetuses**				
*Singleton* (*ref*.)	2948	1.00		
*Multiple*	169	0.00	0.00-	0.995
**Perineal state**				0.818
*Intact* (*ref*.)	2378	1.00		
*Episiotomy*	258	1.73	0.21-14.13	0.607
*Tear*	481	0.79	0.17-3.63	0.757

*Significant predictors.

*Ref*.: *reference categories.*

**Table 7:** Multivariate analyses of predictors of perinatal death

**Variables**	**N**	**AOR**	**95% CI**	**Sig. (2 sided)**
**Age (years)**				0.137
*Adolescent*: *10*-*19*	298	1.43	0.89-2.30	0.141
*Adult*: >*19* (*ref*.)	2797	1.00		
**Work status**				0.674
*Student*	743	0.73	0.30-1.79	0.491
*Unemployed*	97	0.85	0.37-1.97	0.711
*Employed* (*ref*.)	2255	1.00		
**Matrimonial status**				
*Married* (*ref*.)	1453	1.00		
Single*	*1642*	*1.61*	*1.16*-*2.23*	*0.005*
**Referral status**				
Referred case*	*150*	*3.08*	*1.91*-*4.98*	<*0.001*
*Not*-*referred case* (*ref*.)	2945	1.00		
**Gravida**				0.590
*1st pregnancy*	902	1.04	0.71-1.53	0.846
*2*-*4th pregnancy* (*ref*.)	1626	1.00		
≥*5th pregnancy*	568	1.33	0.77-2.30	0.304
**Parity**				0.002
1st delivery*	*116*	*1.90*	*1.11*-*3.42*	*0.019*
*2*-*4th delivery* (*ref*.)	2681	1.00		
≥5th delivery*	*298*	*1.33*	*0.77*-*2.30*	*0.008*
**Gestational age** (weeks)				
<37*	*880*	*4.37*	*3.16*-*6.04*	<*0.001*
*37*- < *42* (*ref*.)	2160	1.00		
≥42*	*55*	*3.94*	*1.81*-*8.55*	*0.001*
**Delivery mode**				
*Vaginal* (*ref*.)	3039	1.00		
*Cesarean*	56	0.44	0.10-1.87	0.265
**Number of fetuses**				
*Singleton* (*ref*.)	2927			
Multiple*	*168*	*0.08*	*0.02*-*0.32*	<*0.001*
**Perineal state**				0.479
*Intact* (*ref*.)	2358	1.00		
*Episiotomy*	258	1.08	0.59-1.95	0.806
*Tear*	479	1.31	0.85-2.03	0.226

*Significant predictors.

*Ref*.: *reference categories.*

## Discussion

The adolescent pregnancy rate (9.3%) in our study differed markedly from that got by Tebeu et al. in 2006 of 26.54% which sampled women in the North of Cameroon [3]. The differences in these rates is probably due to our larger sample size (5997 versus 3328 for the latter) but more so due to the socio-cultural differences between the two settings. The former study (ours) was carried out in an urban and cosmopolitan setting where education of the girl child is promoted compared to the latter study’s setting characterized by rural, indigenous and predominantly Muslim setting where early marriages are encouraged and education of the girl child not considered priority. Ezegwui and collaborators of neighboring Nigeria equally observed a marked difference in the prevalence of adolescent pregnancies between the non-Muslim South and Muslim north of the country (1.67% versus 11.8%) [4].

Referral status rate among adolescent parturients was significantly higher compared to that in their non-adolescent counterparts (6.4% against 4.3%, p = 0.02). In our context, teenagers are often socio-economically disfavored (confirmed by the lower employment rate in the adolescent group compared to the adult group, 44.6%versus75.8%, p < 0.001) (Table 1), dependent and followed up in small poorly equipped health facilities (health centers) incapable of handling complications of pregnancy and delivery especially in teenagers. Studies in other settings concur this and explain the more referrals of adolescents from such centers to bigger and better equipped hospitals like the study setting [4,5].

Our study findings of a single marital status of 79.4% for adolescent parturients were similar to those of the 2011 Demographic Health Survey (DHS) which reported that 73.9% of adolescent mothers were single and had a highly fertility rate of 127 per thousand [2]. Being single an author found is a risk factor for poor outcomes in adolescent pregnancies [6].

The difference in mean parity in the adolescents group compared to the adults group (1.90 ± 0.30 versus 2.08 ± 0.37, p < 0.001) could be explained by the fact that 76.5% of our teenage parturients were primigravida. Ezegwui and collaborators reported a similar primiparous rate of 78.3% among adolescent parturients [4].

The significantly higher rate of preterm deliveries in the adolescent mothers studied compared to adults (Table 2) is a common finding by several other authors [4,7,8]. The other gestational duration extreme (post-term) we observed in adolescents compared to adult parturients was also observed by another author [8]. Higher post-term rates in adolescents compared to their non-adolescent counterparts may be due to hypophyseal as well as uterine immaturity while the higher prematurity rates on the other hand may be due to poor follow-up and probably higher infection rates common in the adolescent compared to adult parturients. Some authors in the USA have however observed that post-term deliveries may be more common in African-American adolescents (of similar origin as our study sample) compared to other races [8,9] especially in those with very little education and if pregnancy occurred while taking oral contraception [8].

The absence of a significant difference in the cesarean delivery rates between adolescent and adult parturients group was contradictory to findings of some authors [4], [9] but similar to that observed by Safid and collaborators [10]. This could be explained by the fact that our study setting is a referral center and as such receives indications for and carries out as many cesarean sections for adolescent as well as non-adolescent parturients. Authors who observed a difference observed higher rates in adolescents compared to adult parturients especially emergency cesarean sections [4,9]. Studies with high rates of cesarean section among adolescents attribute this to biological immaturity [11]. Another study on the other hand revealed that age may not be the cause but rather socioeconomic factors that result in poorer pregnancy follow-up in adolescent mothers compared to adults, and a higher likelihood to end up having a C-section [12]. An author rather reported a significantly higher occurrence in adolescent of Caucasian American origin [9].

There was no significant difference between the adolescent and the adult group with respect to instrumental delivery (Table 3). This is contrary to findings by some authors which suggest it is more common in adolescents compared to adult parturients [4,10,13] and others which suggest the contrary [7,14]. Our findings however concur with those of Scanlon and collaborators who argue that indication for instrumental delivery depends on other factors other than parturient’s age [15]. It was observed however that adolescents required significantly twice more episiotomies than the adult group (OR 2.15 95% CI 1.59-2.90). This is a common finding in literature [4,8]. Pelvic and perineal immaturity can well explain this higher rate of episiotomies in adolescent parturients [11].

The rate of perineal tears in our study surprisingly did not differ between adolescent and adult parturients. However assuming parturients in both groups who had episiotomies would have had tears had the procedure not been carried out (that is episiotomies were tears), the difference in the rate of tears between both groups became significant (30.5 and 23.3% for the adolescent and non-adolescent groups respectively, p = 0.001; OR 1.45 95% CI 1.16-1.82). This underscores the importance of episiotomies in preventing tears.

There was no significant difference between the two groups with respect to perineal tear severity (Table 3). This might be explained by the fact that our study setting is a Teaching Hospital where some deliveries are carried out by students and residents in training with risk of tears even in the adult parturients if the perineum is not well protected during delivery.

There was no significant difference between the two groups as far as blood loss was concerned (Table 3). This finding was also observed by Iacobelli and collaborators [7]. This suggests that bleeding depends more on other causes such as the technical skills of the staff carrying out the delivery, active management of the third stage of labor as well as physiological processes rather than parturient’s age.

The significantly higher odds of apparent and perinatal deaths on controlling for mother’s employment status, referral status, mode of delivery, mode of vaginal delivery and number of gestas (singleton or multiple) we observed in our study: AOR 1.75 95% CI 1.25-2.47 and AOR 1.69 95% CI 1.17-2.45 respectively (Table 4) has been observed by other authors [7,10-13]. Having been referred, primiparous or having had ≥5 deliveries, preterm deliveries, multiple gestation or being a single mother were found to be independent predictors of perinatal mortality (Table 7). These findings are similar to those by other authors in other developing country settings [16-20]. Adolescence was not identified in our study as an independent predictor of perinatal mortality. This is a similar finding to that of a study carried out by Restrepo-Méndez and collaborators [21] in Brazil but contrary to that by Fawole and collaborators [20] in Nigeria. Having been referred ironically was positively associated (AOR 3.08 95% CI 1.91-4.98) with perinatal mortality probably due to late referrals.

The maternal mortality ratio (irrespective of age), MMR, observed in this study (a facility-based) is higher than the National estimates got from community-based surveys: 690 per 100,000 live births [22] but similar to studies in other tertiary institutions in Cameroon and Nigeria [23,24]. It is however 1.46 less than that obtained by Tebeu and collaborators in a secondary-care-facility-based Cameroonian study [25], and 2.76 times less than that of an Indian tertiary facility-based study [26]. Facility-based MMR are generally higher than community-based estimates because of referrals to these structures of more severe cases.

With respect to age, we observed a MMR in adolescent parturients twice less that in their adult counterparts (Table 4). Fawole and collaborators [23] observed adolescence was not an independent predictor of maternal mortality. The higher referral rates of adolescent pregnancies to the study site (a University teaching hospital) compared to pregnancies of their adult counterparts may explain this lower maternal mortality rate unlike the other obstetrical outcomes described earlier.

On multivariate analyses, predictors of maternal death included having been referred, having had 5 deliveries or more and preterm deliveries (Table 6). These were also predictors of perinatal fetal death, as well as were being single mother, being a primip or having a multiple gestation (Table 7). Having been referred was positively associated (AOR 4.60 95% CI 1.37-15.48) with maternal mortality probably due to late referrals. Fawole and collaborators in a similar setting in Nigeria found having had 4 or more deliveries an independent predictor of maternal mortality of deliveries in health facilities [23]. Our study did not identify other independent predictors of in-facility maternal mortality identified by other authors such as having had a cesarean section or being an adolescent [23], [27]. Authors attribute the excess maternal mortality risk in adolescent mothers to socioeconomic factors rather than mother’s age [28]. Socioeconomic status (work status) however was not found to be a predictor in our study (Tables 6 and 7).

In an attempt to determine if pregnancy outcomes differed between parturients in early (10–14 years), middle (15–17 years) or late adolescence (18–19 years), sub-analyses were carried out (Table 5). These sub-analyses revealed no significant differences between the three sub-groups. This finding is similar to those of Leppälahtiand collaborators [29] who but for cesarean section rates, found no significant differences in pregnancy outcomes between parturients in early, middle and late adolescence.

## Conclusions

Adolescent deliveries are common in Cameroon, most of which are from single and unemployed mothers. These deliveries are associated with more referrals and poorer maternal and fetal outcomes compared to pregnancies in adults. Mothers in early adolescence (10–14 years) do not differ significantly in pregnancy outcomes with those in middle (15–17 years) or late adolescence (18–19 years).

Unlike other obstetrical outcomes, maternal mortality among adolescent mothers our study found is lower than in their adult counterparts. Having been referred, having had ≥5 deliveries and preterm deliveries are independent predictors of in-facility maternal mortality as well as of perinatal death alongside being primiparous, and having multiple gestation. Adolescent-specific-and-friendly interventions are needed in Cameroon to prevent adolescent pregnancies and improve their outcomes. This should include among other things early referrals to obstetrics units capable of handling the above mentioned predictors of in-facility maternal and perinatal mortality.

## Competing interests

The authors declare that they have no competing interests.

## Authors’ contributions

FYF conceived the study, participated in the study design, data collection, and drafting and editing of the manuscript. TTY and ENM, participated in the study design, data collection and analyses, drafting and editing of the manuscript. PNN, HJF, JTF and REM contributed to the design of the study and editing of the manuscript. All authors read and approved the final manuscript.

## Pre-publication history

The pre-publication history for this paper can be accessed here:

http://www.biomedcentral.com/1471-2393/14/102/prepub

## References

[B1] The WHO study group on young people and health for all by the year 2000 Young People’s Health-a Challenge for Society 1986 No. 73 1 Geneva: World Health Organization, WHO technical report series 3085358

[B2] Measure DHS, ICF International Calverton Demographic Health Survey Cameroon 2011 2011 Maryland, U.S.A: Measure DHS, ICF International Calverton

[B3] Tebeu PM Tantchou J Obama Abena MT Mevoula Onala D Leke RJ Delivery outcome of adolescents in Far North Cameroon Rev Med Liege 2006 66 2 124 127 16566121

[B4] Ezegwui HU Ikeako LC Ogbuefi F Obstetric outcome of teenage pregnancies at a tertiary hospital in Enugu Nigeria. Niger J Clin Pract 2012 15 2 147 150 Doi: 10.4103/1119-3077.97289 10.4103/1119-3077.97289 22718161

[B5] The Alan Guttmacher Institute Teen sex and pregnancy 1999 New York: Alan Guttmacher Institute

[B6] Obare F van der Kwaak A Birungi H Factors associated with unintended pregnancy, poor birth outcomes and post-partum contraceptive use among HIV-positive female adolescents in Kenya BMC Women’s Health 2012 12 34 23039966 10.1186/1472-6874-12-34 PMC3492047

[B7] Iacobelli S Robillard PY Gouyon JB Hulsey TC Barau G Bonsante F Obstetric and neonatal outcomes of adolescent primiparous singleton pregnancies: a cohort study in the South of Reunion Island, Indian Ocean The J of Maternal-Fetal and Neonatal Medicine 2012 25 12 2591 2596 10.3109/14767058.2012.718003 22889253

[B8] Harville EW Madkour AS Xie Y Predictors of birth weight and gestational age among adolescents Am J Epidemiol 2012 176 Suppl S150 S163 23035139 10.1093/aje/kws231 PMC3530360

[B9] Penfield CA Cheng YW Caughey AB Obstetric outcomes in adolescent pregnancies: a racial/ethnic comparison Matern Fetal Neonatal Med 2013 1 5 Early Online 10.3109/14767058.2013.784738 23488933

[B10] Safid BA Catalmo MM Dieker IJ Mann LI Birth to teenagers Obstet Gynecol 1996 87 668 689 8677065 10.1016/0029-7844(96)00007-5

[B11] Mahavarkar SH Madhu CK Mule VD A comparative study of teenage pregnancy J Obstet Gynecol 2008 28 6 604 607 10.1080/01443610802281831 19003655

[B12] Raatikainen K Heiskanen N Verkasalo PK Heinonen S Good outcome of teenage pregnancies in high-quality maternity care Eur J Public Health 2006 16 157 161 16141302 10.1093/eurpub/cki158

[B13] Felice ME Feinstein RA Fisher MM Kaplan DW Olmedo LF Rome ES Staggers BC Adolescent pregnancy - Current trends and issues: 1998 American Academy of Pediatrics Committee on Adolescence, 1998–1999 Pediatrics 1999 103 516 520 9925856 10.1542/peds.103.2.516

[B14] Brown HL Fan YD Gonsoulin WJ Obstetric complications in young teenagers South Med J 1991 84 46 48 1986428 10.1097/00007611-199101000-00012

[B15] Scanlon KS Yip R Schieve LA Cogswell ME High and low hemoglobin levels during pregnancy: differential risks for preterm birth and small for gestational age Obstet Gynecol 2000 96 741 748 10.1016/S0029-7844(00)00982-0 11042311

[B16] Bayou G Berhan Y Perinatal mortality and associated risk factors: a case control study Ethiop J Health Sci 2012 22 3 153 162 23209349 PMC3511893

[B17] Andargie G Berhane Y Worku A Yigzaw K Predictors of perinatal mortality in rural population of Northwest Ethiopia: a prospective longitudinal study BMC Public Health 2013 13 168 10.1186/1471-2458-13-168 23433304 PMC3599850

[B18] Vaahtera M Kulmala T Ndekha M Koivisto A Cullinan T Salin M Ashorn P Antenatal and perinatal predictors of infant mortality in rural Malawi Arch Dis Child Fetal Neonatal Ed 2000 82 F200 F204 10.1136/fn.82.3.F200 10794786 PMC1721093

[B19] Ali AA Elgessim ME Taha E Adam GK Factors associated with perinatal mortality in Kassala, Eastern Sudan: a community-based study 2010–2011 J Trop Pediatr 2013 [Epub ahead of print] 10.1093/tropej/fmt075 24052575

[B20] Fawole AO Shah A Tongo O Dara K El-Ladan AM Umezulike AC Alu FE Eniayewun AB Fabanwo AO Adewunmi AA Adegbola O Adebayo AA Obaitan FO Onala OE Usman Y Sullayman AO Kailani S Sa’id M Determinants of perinatal mortality in Nigeria Int J Gynaecol Obstet 2011 114 1 37 42 doi: 10.1016/j.ijgo.2011.01.013. Epub 2011 Apr 12 10.1016/j.ijgo.2011.01.013 21489535

[B21] Restrepo-Méndez MC Barros AJ Santos IS Menezes AM Matijasevich A Barros FC Victora CG Childbearing during adolescence and offspring mortality: findings from three population-based cohorts in southern Brazil BMC Public Health 2011 11 781 10.1186/1471-2458-11-781 21985467 PMC3207956

[B22] World Health Organization United Nations Children’s Fund, United Nations Population Fund and the World Bank, Trends in Maternal Mortality: 1990–2010, Estimates developed by WHO, UNICEF, UNFPA and the World Bank, 2012. Consulted on 15.08 2013 http://www.unfpa.org/webdav/site/global/shared/documents/publications/2012/Trends_in_maternal_mortality_A4-1.pdf

[B23] Fawole AO Shah A Fabanwo AO Adegbola O Adewunmi AA Eniayewun AB Dara K El-Ladan AM Umezulike AC Alu FE Adebayo AA Obaitan FO Onala OE Usman Y Sullayman AO Kailani S Sa’id M Predictors of maternal mortality in institutional deliveries in Nigeria Afr Health Sci 2012 12 1 32 40 23066417 PMC3462508

[B24] Mbassi SM Mbu R Bouvier-Colle MH Use of routinely collected data to assess maternal mortality in seven tertiary maternity centers in Cameroon Int J Gynaecol Obstet 2011 115 3 240 3 doi: 10.1016/j.ijgo.2011.07.018 10.1016/j.ijgo.2011.07.018 21930267

[B25] Tebeu PM Ngassa P Kouam L Major AL Fomulu JN Maternal mortality in Maroua Provincial Hospital, Cameroon (2003–2005) West Indian Med J 2007 56 6 502 7 18646493

[B26] Guin G Sahu B Khare S Kavishwar A Trends in Maternal Mortality and Impact of Janani Suraksha Yojana (JSY) on Maternal Mortality Ratio in a Tertiary Referral Hospital J Obstet Gynaecol India 2012 62 3 307 11 doi: 10.1007/s13224-012-0221-1 10.1007/s13224-012-0221-1 23730035 PMC3444551

[B27] Gupta SD Khanna A Gupta R Sharma NK Sharma ND Maternal mortality ratio and predictors of maternal deaths in selected desert districts in Rajasthan: a community-based survey and case control study Womens Health Issues 2010 20 1 80 5 Doi: 10.1016/j.whi.2009.10.003 10.1016/j.whi.2009.10.003 20123178

[B28] Ali M Lulseged S Factors influencing adolescent birth outcome Ethiop Med J 1997 35 1 35 42 9293145

[B29] Leppälahti S Gissler M Mentula M Heikinheimo O Is teenage pregnancy an obstetric risk in a welfare society? A population-based study in Finland, from 2006 to 2011 BMJ Open 2013 3 e003225 Doi: 10.1136/ bmjopen-2013-003225 23959755 10.1136/bmjopen-2013-003225 PMC3753503

